# Pleiotropic Effects of IL-33 on CD4^+^ T Cell Differentiation and Effector Functions

**DOI:** 10.3389/fimmu.2019.00522

**Published:** 2019-03-20

**Authors:** Fernando Alvarez, Jörg H. Fritz, Ciriaco A. Piccirillo

**Affiliations:** ^1^Department of Microbiology and Immunology, McGill University, Montréal, QC, Canada; ^2^Program in Infectious Diseases and Immunology in Global Health, Centre for Translational Biology, The Research Institute of the McGill University Health Center, Montréal, QC, Canada; ^3^Centre of Excellence in Translational Immunology, Montréal, QC, Canada; ^4^McGill University Research Center on Complex Traits, McGill University, Montréal, QC, Canada

**Keywords:** T cell differentiation, Th17 and Tregs cells, th1/th2 balance, infection, immunoregulation, IL-33, ST2

## Abstract

IL-33, a member of the IL-1 family of cytokines, was originally described in 2005 as a promoter of type 2 immune responses. However, recent evidence reveals a more complex picture. This cytokine is released locally as an alarmin upon cellular damage where innate cell types respond to IL-33 by modulating their differentiation and influencing the polarizing signals they provide to T cells at the time of antigen presentation. Moreover, the prominent expression of the IL-33 receptor, ST2, on GATA3^+^ T helper 2 cells (T_H_2) demonstrated that IL-33 could have a direct impact on T cells. Recent observations reveal that T-bet^+^ T_H_1 cells and Foxp3^+^ regulatory T (T_REG_) cells can also express the ST2 receptor, either transiently or permanently. As such, IL-33 can have a direct effect on the dynamics of T cell populations. As IL-33 release was shown to play both an inflammatory and a suppressive role, understanding the complex effect of this cytokine on T cell homeostasis is paramount. In this review, we will focus on the factors that modulate ST2 expression on T cells, the effect of IL-33 on helper T cell responses and the role of IL-33 on T_REG_ cell function.

## Multi-Faceted Functions of IL-33

Barrier sites are exposed to varying levels of danger at every moment, which requires the constant involvement of the local immune system to maintain epithelial function and immune homeostasis. As such, many foreign and self-derived warning signals dictate the response of these immune cells. The molecules that provide these signals are classified as pathogen-associated molecular patterns (PAMPs) or danger-associated molecular patterns (DAMPs). However, some specialized endogenous molecules, released upon cellular damage, were improperly organized using these definitions. Thus, a new concept was introduced during the *EMBO Workshop on Innate Danger Signals and HMGB1* in February 2006, which would separate PAMPs from self-signals. Joost Oppenheim introduced at that meeting what he coined “alarmins,” self-molecules released upon cellular damage that play a role in modulating the immune response (1, 2). The proposed description classifies “alarmins” as molecules that (1) are released upon non-programmed cells death; (2) can be produced by immune cells without dying; (3) can recruit and activate receptor-expressing immune cells; and (4) can contribute to the restoration of immune homeostasis and epithelial repair mechanisms (1). In recent years, several examples of dysregulated expression or activity of alarmins were associated with immune-related pathologies in many diseases. Thus, alarmins can play pro-inflammatory or regulatory roles at the site of inflammation (3).

Of the many members of alarmins, the IL-1 family, comprised of 11 members, was introduced early in this classification (4). IL-1 family members include IL-1α, IL-1β, IL-18, IL-33, IL-36α, IL-36β, IL-36γ, and IL-37 which possess agonist properties and IL-1Ra, IL-36Ra, and IL-38, which possess antagonist properties on their respective receptors (5). A unique feature of this family, with the exception of IL-1Ra, is their capacity to accumulate as pro-cytokines and possess enzymatic cleavage sites in their sequence (6). However, cleavage is not always required for these pro-cytokines to bind and activate their respective receptors. For example, as caspase 1 and caspase 8 are required for the activation of IL-1β and IL-18, pro-IL-33 does not require enzymatic processing to exert its biological activity (6). However, processing by neutrophils proteases, notably cathepsin G and elastase, and proteases brought by airway allergens were shown to enhance IL-33 activity (6, 7). This peculiarity reveals that IL-33, as opposed to IL-1β or IL-18, exerts most of its effect in a caspase-independent manner (6). Thus, IL-33 possesses intrinsic biomolecular peculiarities that dictate its role at mucosal sites and its effect on the innate and adaptive immune system.

Expression of ST2 was first described in CD4^+^ T_H_2 cells (8). However, a wide range of immune cells has been described to respond to IL-33 directly. A functional ST2 receptor was notably described in eosinophils (9), basophils (10), natural killer (NK), and NK-T cells (11, 12), as well as group 2 innate lymphoid cells (ILC2s) (13). In eosinophils, IL-33 was shown to directly facilitate their maturation through enhanced survival, activation and adhesion (14). Similarly, IL-33 potentiates adhesion and histamine release in basophils (15). IL-33 is also known to facilitate the maturation, migration from the bone marrow and local functions of ILC2s in the lungs (13, 16). Furthermore, dendritic cells (DCs) can respond to IL-33 directly to polarize naïve T cells into T_H_2 or facilitate T_REG_ proliferation (17, 18). Interestingly, although the effect of IL-33 was originally thought to be a determinant of type 2 immune responses, it was shown to also favor the expansion of NK and NK T cells during viral infections (11, 12). Thus, IL-33 has pleiotropic functions in directing the innate immune response, a feature that is also found in its effect on adaptive immunity, most notably in the function and differentiation of CD4^+^ T cells.

In mammals, T cells are critical members of the immune system and play a pivotal role in all aspects of immune responses from the effective clearance of pathogens to the establishment of a memory response and the quick return to immune homeostasis. CD4^+^ T cells are characterized by their ability to recognize antigens through their T cell specific receptor (TCR), upon which they undergo rapid clonal expansion and differentiate into functionally distinct T_H_ subsets. These subsets then migrate and orchestrate the immune response at inflammatory sites. It is of no surprise that the distinct subsets of helper CD4^+^ T cells, T_H_1, T_H_2, and T_H_17 cells, respond to alarmins of the IL-1 family in order to proliferate and function locally (19). However, the categorization of T cells by their master transcription factors like T-bet, GATA3, RORγT or Foxp3, does not reflect the high level of T cell plasticity observed *in vivo*. For example, T cells expressing both GATA3 and T-bet were observed in the lung during infection with parasites (20). Similarly, although ST2 is strongly associated with the function of T_H_2 T cells (8), T_H_1 cells can also transiently express it (21). Moreover, it was shown that Foxp3^+^ T_REG_ cells and T_H_17 cells signal through IL-33 to modulate their respective functions (22, 23).

In this review, we will focus on the effects of IL-33 on CD4^+^ T cell responses. We will highlight recent advances in our understanding of the IL-33 pathway and its impact on T cell differentiation and effector functions, including the modulatory role of IL-33 on Foxp3^+^ T_REG_ cells, in both autoimmune and infectious diseases.

## Regulation of IL-33 Expression and Secretion

IL-33 is constitutively expressed as a nuclear protein in epithelial and endothelial cells. Body-wide analysis through immunohistochemistry, mRNA transcripts and a unique *il-33-LacZ* reporter mouse line revealed that IL-33 is constitutively expressed in secondary lymphoid tissues, but more prominently found at mucosal sites like the gut and lungs, as well as in the brain and adipose tissues (24). However, although humans and mice share most of the constitutive expression of IL-33, species-specific differences exist. For example, it was shown that murine keratinocytes express IL-33 constitutively whereas human keratinocytes required prior IFNγ stimulation (25). Thus, conclusions derived from mouse models must be corroborated with human samples.

Many biological mechanisms regulate the half-life and activity of IL-33. On one hand, pro-IL-33, a 31 kDa protein, does not require enzymatic cleavage to exert its biological functions, although these can be potentiated by the action of self and non-self proteases and elastases that cut it down to a more potent 20 kDa protein (6, 7, 26). On the other hand, the activity of IL-33 is known to be reduced by: (1) cleavage of IL-33 after Asp178 by caspases 3 and 7 (27); (2) upregulation of the LMP2 proteasome by IFNγ during type 1 immune responses (28); (3) extracellular cysteine oxidation that cause the formation of two disulfide bridges on IL-33 and disrupts its binding to ST2 (29), and (4) the extracellular release of the soluble ST2 (sST2), that acts as a decoy receptor for IL-33 (30, 31). Furthermore, IL-33 lacks a conventional signal sequence or any non-canonical export pathway and thus requires either cellular death by necrosis or necroptosis of endothelial and epithelial cells or a still unknown excretory mechanism by innate immune cells to be released in the extracellular milieu (5, 30). In fact, the full-length IL-33 was shown to bind to chromatin causing it to be 10 times slower than IL-1α (32). This novel post-translational mechanism of cytokine release, along with the many enzymatic and environmental processes described, reveals the fine control of the activity of IL-33 at mucosal surfaces and illustrates the evolutionary control of these immunomodulatory signals.

## IL-33 Signaling

ST2 was first described as an orphan receptor until the discovery of IL-33 (31). A member of the Toll-like/Interleukine-1 receptor superfamily, it was shown that it forms a heterodimer with the ubiquitous IL1R accessory protein (IL1RAcp) at the membrane surface in order to bind IL-33. Interestingly, all the members of the IL-1 family share a common intracellular Toll/IL-1 receptor (TIR) domain. However, four distinct isoforms of ST2 were described: (1) the membrane-bound ST2 (ST2L or ST2), which provides the activation pathway; (2) the soluble ST2 (sST2)–that originates from another promoter region of the *il1rl1* gene and lacks the transmembrane and cytoplasmic domains of ST2–acts as a decoy for IL-33, and is notably used as a biological marker of cardiac injury (31, 33); the latter two forms are splice variants identified in a tumor cells line 3) ST2V (34), which possesses a hydrophobic tail at the C-terminal; and 4) in chicken, ST2LV (35), which lacks the transmembrane domain of ST2 and whose function remains to be elucidated.

IL-33 binds specifically to ST2, which in turn associates to the IL1RAcP to form a heterodimeric receptor that leads to the dimerization of the TIR domain with the TIR domain of cytosolic adaptor protein myeloid differentiation factor 88 (MyD88). In turn, the N-terminal death domain (28) of MyD88 recruits the IL-1-associated kinase 1 (36) and 4 (37). The IRAK1/4 complex can then activate the downstream mitogen-activated protein kinase (MAPK) through the TNF receptor-associated factor 6 (TRAF6). TRAF6 does not possess enzymatic activity but plays a critical role through its ubiquitin E3 ligase (38). TRAF6 is thus required for the induction of several kinase cascades such as NF-kB, JNK, p38, and PI3K. Interestingly, IL-33 can activate ERK even in TRAF6-deficient cells, indicating a parallel activation cascade upon signaling (38). In fact, IL-33 could still induce the expression of ST2L in TRAF6-deficient embryonic fibroblasts (38), indicating the presence of distinct pathways in the IL-33 cascade. However, most of these analyses were conducted using non-T cell lines, and studies in primary immune cells are warranted (39, 40).

### TRAF6 Activation in T Cells

In T cells, TRAF6 is known to regulate TCR signaling via ubiquitination at Lys(88) of the LAT adapter and phosphorylation of the IKK/NEMO complex (41). Interestingly, TRAF6 deficiency leads to a hyperactivation of the PI3K-AKT pathway in T cells and to T_H_2 polarization in mice (42). Furthermore, TRAF6 is essential for the survival and proliferation of T_REG_ cells that suppress T_H_2 type autoimmunity (43, 44). As such, TRAF6 is required for the maintenance of peripheral tolerance and control of T cell hyper-reactivity. The downstream targets of TRAF6 include the phosphorylation of JNK1/2 (38). JNK1/2 activation is required for T cell differentiation, but not activation, as the lack of JNK leads to a decrease in inflammatory cytokine production, but not proliferation or IL-2 production (45). In fact, the p38-MAPK pathway plays a non-redundant role on memory ST2^+^ T_H_2 cells, since selective inhibition of p38, but not JNK, PI3K or ERK, leads to a decrease in IL-5 production in these cells upon IL-33 stimulation (46). Thus, although TRAF6 deficiency leads to increased T_H_2 differentiation and a lack of T_REG_-mediated suppression, IL-33 signaling is required for T_H_2 function, illustrating the complexity of this signal in T cells.

### ERK Activation in T Cells

Biochemical dissection of the IL-33/ST2 pathway in mammalian cell lines was performed using data mined through an extensive survey of the literature (40). This model includes the phosphorylation and activation of ERK1/2, JNK1/2, p38, and PI3K/AKT downstream of IL-33. However, the underlying processes affected by these changes remain unknown. This is likely due to the large heterogeneity of the recipient cells and their varied epigenetic status. In T cells, ERK activity is notably linked to a reduction in the TCR activation threshold, as it delays the binding of the inhibitory protein SHP-1 to the complex, leading to the activation of T cells under suboptimal stimulation (47). ERK1 is particularly required for T_H_2 but not T_H_1 proliferation and function and plays a major role in a model of experimental asthma (48). On the other hand, lack of ERK2 inhibits T_H_1 and T_H_17 T cell differentiation and function (49, 50). This was shown to occur notably through the control of the master transcription factors of these subsets, as ERK2 suppresses the transcription of Foxp3 (T_REG_) and GATA3 (T_H_2) and favors the expression of T-bet (T_H_1) (49). Interestingly, although the lack of either ERK2 or ERK1 does not hinder the suppressive ability of T_REG_ cells (49), it favors the TGFβ-mediated induction of Foxp3 (50). Thus, ERK1/2 activation is a major pathway involved in the control of the function of T_H_1, T_H_17, T_H_2, and T_REG_ cells at mucosal sites. Further investigation into the T cell-intrinsic modulation of ERK1 and ERK2 by IL-33 might reveal how the distinct T cell subsets respond to this alarmin.

On the other hand, p38, composed of four known members (α, β, γ, δ), plays key roles in T cell activation and proliferation. Constitutive activation of p38α and p38β (p38αβ^Y323F^) was shown to skew T cell differentiation toward T_H_1 and T_H_17 cells (51), whereas knock-down of p38 α/β led to increased T_REG_ cells (52). Interestingly, the IL-33-p38 pathway was shown to be directly linked to the function of ST2^+^ T_H_2 cells, as inhibition of p38-MAPK, but not JNK or PI3K, resulted in a lack of IL-5 production by T_H_2 cells upon IL-33 stimulation (46). Finally, although we know little about the role of JNK activation by IL-33 on T cells, JNK1/2 was shown to play a critical role in T cell function but not activation (45).

While some signaling pathways downstream of IL-33 are known, the transcriptional targets downstream of IL-33 depend largely on the state of the recipient T cell and the environmental context. Thus, in order to fully understand the role of IL-33 on T cells, assessing the effects of IL-33 on the functions of T_H_ cell subsets is required.

## Effect of IL-33 on T_H_ Cell Responses

### Regulation of ST2 Expression

**Figure 1:**
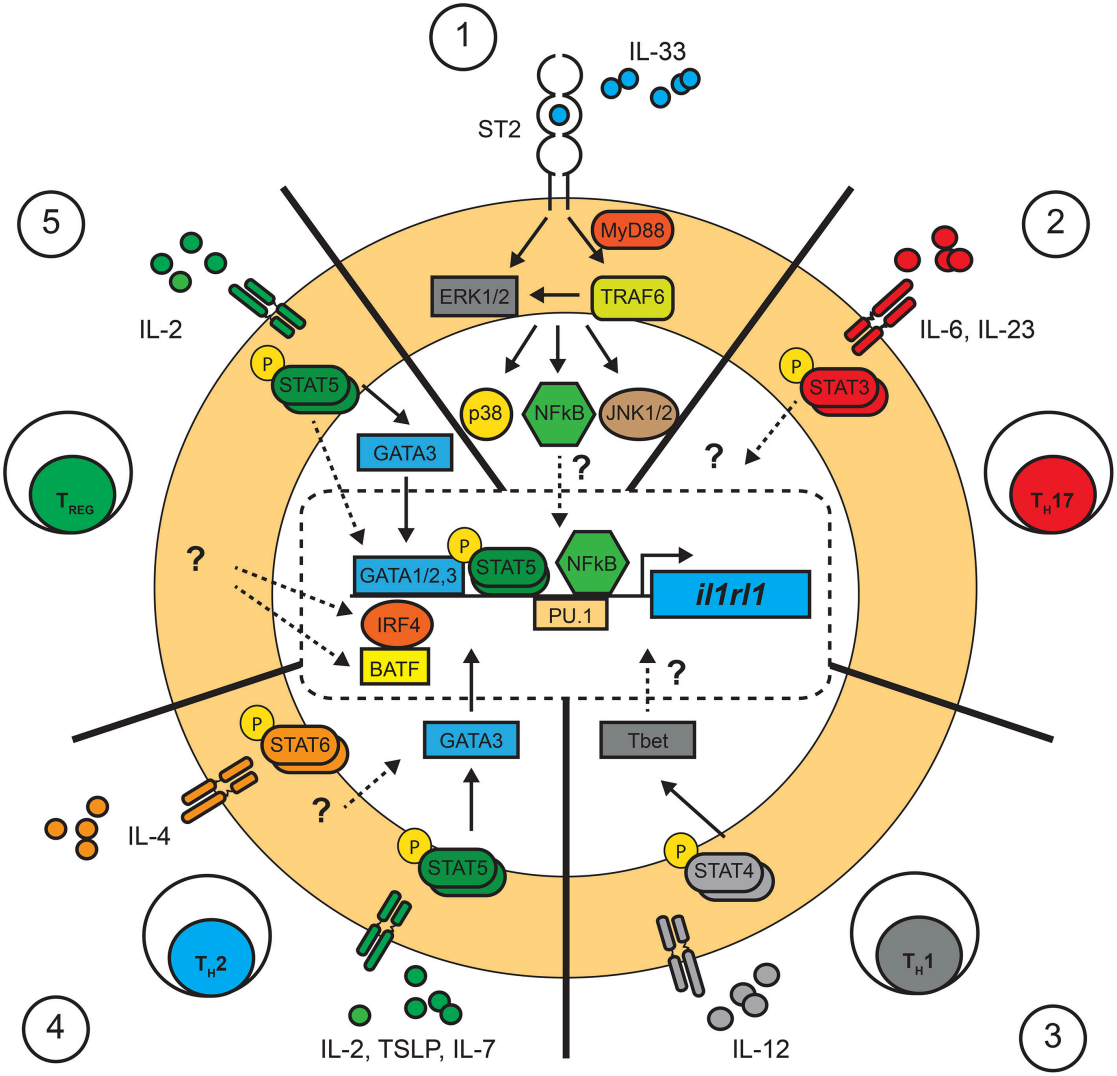
Pathways involved in the control of *il1rl1* (ST2) transcription in T cells. Summary of the transcription factors confirmed or suggested to interact with the promoter region of *il1rl1* (ST2) in T cells. Confirmed (full lines), suggested (dotted lines) and unknown (?) pathways involved in the control of *il1rl1* in T cells. **(1)** IL-33 is required for the maintained expression of ST2 in T cells in T_H_2, T_REG_, and T_H_1 through unknown mechanisms. This process has been suggested to require the involvement of NF-κB translocation and its binding to a consensus sequence in the promoter region of *il1rl1*. **(2)** T_H_17 cells: The molecular pathways involved in the expression of ST2 in T_H_17 T cells remains largely unknown, although the transcription factor STAT3 was shown to bind the promoter region of *il1rl1* in fibroblast cell lines. **(3)** T_H_1 cells: Expression of ST2 in T_H_1 cells was shown to be dependent on a STAT4 signal leading to T-bet expression, although the molecular interaction with *il1rl1* remains unknown. **(4)** T_H_2 cells: It has been suggested that expression of ST2 requires STAT5 signals through the upregulation of GATA3 in conjunction with IL-33 stimulation. Although a STAT6 signal is not necessary, little is known about its role in the maintenance of ST2. **(5)** T_REG_ cells: Expression of ST2 on T_REG_ cells follows a similar pathway as in T_H_2 cells, requiring a STAT5 signal and IL-33 activation for the upregulation of GATA3 and ST2. The transcription factors IRF4 and BAFT were also shown to promote expression of ST2 by T_REG_ cells although little is known about the upstream signals involved.

In an early study, inflammatory factors such as tumor necrosis factor (TNF), IL-1α, IL-1β or Phorbol 12-myristate 13-acetate were shown to be required for the upregulation of the membrane-bound ST2 on responding cells (53). T_H_2 cells were the first to be shown to express ST2 (8). T_H_2 polarizing conditions, involving both STAT5 (IL-2) and STAT6 (IL-4) activation, were shown to induce ST2 on T cells *in vitro*, although multiple rounds of polarization were required (54). In fact, the transcription factor GATA3, associated with the development and function of T_H_2 cells, was necessary for the selective upregulation of ST2 *in vitro*, as genetic deficiency of GATA3 abrogated ST2 expression in T_H_2 cells (55). GATA3 binds an enhancer region situated 12kb up-stream of the transcription start site of *il1rl1* (ST2) (55, 56), a finding confirmed through genome-wide mapping of GATA3 binding (57). Under the same conditions, the expression of ST2 was also dependent on the binding of STAT5 to the intron 7 of *il1rl1* (55) which also leads to the production of IL-13 and IL-5, but not IL-4, *in vitro*, suggesting that STAT5-activating signals, such as IL-2, IL-7 or TSLP are required for the upregulation of ST2 in T _H_2 cells (Figure 1).

Interestingly, the expression of ST2 is particularly enhanced by the provision of exogenous IL-33 in CD4^+^ T cell cultures, illustrating that IL-33, in a positive feedback loop, is directly involved in the up-regulation of its own receptor (55). It has been suggested that IL-33 potentiates STAT5 signaling in T cells since *in vitro* polarized T_H_2 cells show increased STAT5 phosphorylation when exposed to IL-33 (55). On the other hand, a consensus site for NF-κB was found in the *Il1rl1* promoter region (58), revealing a potential mechanism by which IL-33 could regulate its own expression. Nonetheless, further investigations are required in order to understand why and how IL-33 is required for the expression of its own receptor. Thus, both a STAT5 signal (IL-2, IL-7 or TSLP) and IL-33 are sufficient to upregulate ST2 on T_H_2 cells (55) and T_REG_ cells (23) (Figure 1).

The involvement of a STAT6 signal in the development of ST2^+^ T_H_2 cells remains to be understood. In early experiments, IL-4 was required for the polarization of T_H_2 cells, and thus was involved, amid indirectly, in the cells' responsiveness to IL-33. On a molecular level, STAT6 is not known to bind the promoter region of ST2 but does bind to the distal promoter of *gata3* (59). Yet, STAT6, but not GATA3, is necessary for binding the locus control region (LCR) inside the T_H_2 cytokine gene cluster of *il4, il5*, and *il13* (60). As such, STAT6 remodels the LCR, whereas GATA3 acts as a local promoter of these genes. Nonetheless, forced expression of a constitutively activated form of STAT5A (STAT5A1^*^6) through retroviral transduction in T cells revealed that a STAT6 signal was not essential to differentiate T_H_2 cells (61). The binding sites of STAT5 on the gene cluster differs from STAT6 and could illustrate a parallel evolutionary mechanism in the polarization of T_H_2 cells (61). Interestingly, even in these conditions, co-expression of a constitutive GATA3 potentiated the effect of STAT5 in T_H_2 cell development (61). Thus, although STAT5 plays a significant role, a co-stimulatory STAT6 signal is required to potentiate GATA3 expression and leads to the full differentiation of T_H_2 cells.

Apart from GATA3, other transcription factors were shown to bind to the distal promoter site of *il1rl1* (ST2). Four GATA1 binding sites were identified within 1,001 bp of the distal promoter region of *il1rl1* in human and murine cells lines (62, 63). GATA2 and PU.1 were further identified to exert key roles in the expression of ST2 in mast cells and basophils as they bind the distal promoter region of the *il1rl1* gene (64, 65). Interestingly, while GATA1 acted as a repressor, GATA2 provided a transactivation signal for the expression of *il1rl1* (64). Little is known as to the role of GATA1 and GATA2 in the later stages of T cell polarization and function. A report demonstrated that GATA1 possesses a degree of redundancy with GATA3 in T cells, as it suppresses T_H_1 differentiation and functions in a similar, yet less efficient, manner as GATA3 (66). More recent evidence points to a possible role of PU.1 in the regulation of GATA3 expression in T cell differentiation. PU.1 is required for the development of T cells in the thymus (67), and is expressed in T_H_9 and not in T_H_1 cells (68). Interestingly, PU.1 can alter GATA3 promoter regions in dendritic and T cells and was found to facilitate the expression of *il5* and *il13*, but not *il4* (68, 69). Although yet unknown, the role of PU.1 in ST2^+^ T_H_2 might reveal why these cells respond to IL-33 by expressing IL-5 and IL-13, but not IL-4 (55). A recent report identifies the transcription factors IRF4 and BATF as binding the *il1rl1* loci in T_REG_ cells (70) (Figure 1). In fact, a reduced expression of BATF lead to a decrease in ST2 expression in T_REG_ cells (71). Thus, T_REG_ cells may possess distinct mechanisms to control the transcription of *il1rl1*.

Surprisingly, ST2 can be transiently expressed by T_H_1 cells (21). In these reports, the upregulation of ST2 was significantly lower and short lived when compared to ST2^+^ T_H_2 cells and was dependent on the expression of the transcription factor T-bet and the IL-12-dependent STAT4 signal (21, 72). Interestingly, co-stimulation with IL-33 was also required for the expression of ST2. Although these observations were corroborated *in vivo* with STAT4^−/−^ and Tbet^−/−^ mice during the course of a lymphocytic choriomeningitis virus (LCMV) infection (21), it was suggested that these cells might represent a hybrid T-bet^+^GATA3^+^ cell subset as low levels of GATA3 were found to be upregulated in a subset of Tbet^+^ cells (20, 56). Nonetheless, further investigations are required to understand the transcriptional mechanisms by which T_H_1 cells express ST2.

Finally, T_H_17 cell, expressing the transcription factor RORγT and producing IL-17A, could, under strong TCR stimuli, express ST2 in the small intestine (22). The process by which T_H_17 cells upregulate ST2 remains unclear. However, it is well-known that STAT3 signaling plays a major role in the development and cytokine expression of T_H_17 T cells (73). Recently, it was show that STAT3, along with ERK, had the potential to upregulate the proximal promoter region of ST2 in both human and murine fibroblastic cells lines (74). Although the proximal region of ST2 results in the truncated soluble form of ST2 (sST2), an analog mechanism involving the distal promoter might be found in ST2^+^ T_H_17 cells.

### T_H_2 Cell Development and Function

Since the discovery of IL-33, progress has been made to identify its multifunctional roles. Initially, IL-33 was described for its role in promoting type 2 immunity in infectious and allergic diseases (75). Polymorphisms in the *il1rl1* or *il33* genes are found in patients suffering from exacerbated type 2 immune responses, notably severe atopic dermatitis and asthma, illustrating the important role of these genes in the susceptibility to allergic diseases (36, 76). IL-33 administration in the airways of mice enhances T_H_2-associated cytokine production in the lungs, increases mucus production and causes a severe type 2 airway hyper-reactivity that mimics the pathophysiology of asthma (37). These reports highlight the role of IL-33 in the differentiation, and function of T_H_2 cells (77–79).

Naïve T cell express little to no ST2 on their surface. ST2 is expressed *in vitro* when T cells receive TCR activation in combination with cytokine polarization that drives T_H_2 T cell differentiation (55, 80). Thus, unique to T cells, TCR engagement is, along with STAT5 and IL-33, a critical signal for naïve T cells to upregulate the ST2 receptor. Conversely, differentiated T_H_2 cells maintain the ability, long after TCR stimuli, to respond to IL-33 and produce IL-5 and IL-13 (55). Thus, T cells must undergo a round of activation to upregulate the receptor but, once activated remain capable of responding to IL-33. Human CD4^+^ T cells also express the ST2 receptor *in vitro* upon T_H_2, but not T_H_1, differentiation, although IL-4, a STAT6 inducer, was used in these assays (81).

**Figure 2:**
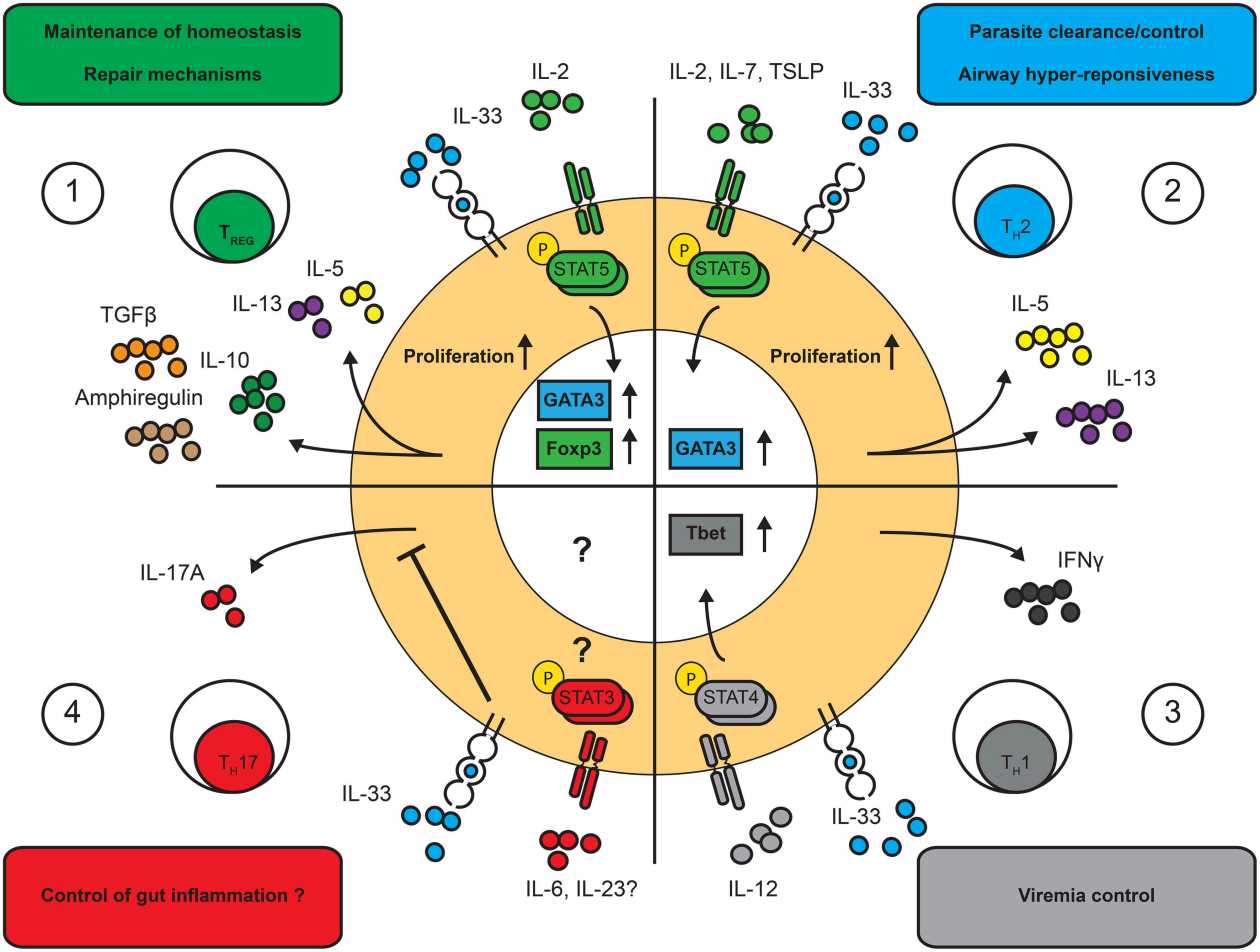
Effects of IL-33 on T cell functions. IL-33 is a multi-faceted cytokine regulating distinct T cell functions and in a highly context-dependent manner. Known functional outcomes of IL-33 on T cell driven immune responses. **(1)** T_REG_ cells: IL-33 increases proliferation of T_REG_ cells and facilitates the production of amphiregulin, IL-10 and TGFβ as well as low levels of IL-5 and IL-13 in a STAT5-dependent manner. **(2)** T_H_2 cells: IL-33 enhances the proliferation and the expression of IL-5 and IL-13 in T_H_2 cells in a STAT5-dependent manner. **(3)** T_H_1 cells: IL-33 was shown to enhance IFNγ production in T_H_1 cells in a STAT4-dependent manner. **(4)** T_H_17 cells: IL-33 was shown to inhibit IL-17 production in T_H_17 cells. The effects on other T cell functions remains to be assessed.

*In vitro*, IL-33 enhances IL-5 and IL-13 production, but not IL-4, in T_H_2 polarized cells (55, 80). This phenotype is unusual, as IL-4 expression was long thought to be the hallmark of differentiated T_H_2 cells (82) and hinted that *in vitro* IL-33-responding T_H_2 cells may have undergone further epigenetic modifications (83). In contrast, IL-33 administration leads to the accumulation of IL-4^+^ T_H_2 cells in the lungs and lymph nodes of treated mice (37, 84). This discrepancy could be due to the effect of IL-33 on APCs, directly involved in T cell differentiation. In fact, IL-33 modulates the differentiation and maturation of DCs as they polarize naïve T cells into ST2^+^ T_H_2 cells (18). Similarly, IL-33, in conjunction with TGFβ, can facilitate IL-9 production in both mouse and human T cells (85, 86). Thus, the effect of IL-33 signaling on the cytokine production of T cells is highly dependent on the cytokine microenvironment (Figure 2).

IL-33 plays an important role in the pathology of asthma and the T_H_2 cell differentiation *in vivo*. Immunization of mice to a single dose of ovalbumin (OVA) (5) together with IL-33 induces long-lasting memory T_H_2 cells that leads to severe asthma-like pathology in the lungs. These IL-33-induced OVA-specific T_H_2 cells produce particularly high levels of IL-5 and IL-13 upon re-stimulation with OVA, a phenomenon not seen in memory T_H_2 cells of mice were immunized with OVA alone (84). Furthermore, when mice are exposed to airway antigens, ST2^−/−^ T_H_2 cells produce less IL-13, while ST2^−/−^ ILC2 functions remain unaffected (87). Concomitantly, memory IL-5–secreting ST2^+^ T_H_2 cells have been isolated from patients suffering from eosinophilic chronic rhinosinusitis, a common allergic condition (46).

However, once ST2^+^ T_H_2 cells are developed, unexpected outcomes have been observed in response to IL-33. When *in vitro* polarized OVA-specific, ST2-deficient (OVA-Tg/ST2^−/−^) or WT (OVA-Tg) TH2 cells are donor ST2^−/−^ T_H_2, not WT T_H_2, cells expressed higher levels of IL-5 production concomitant with a more severe cellular infiltrations in the lungs (88). Similarly, when ST2^−/−^ mice were exposed to extracts containing ragweed, dust mite and *Aspergillus fumigatus*, a more severe form of airway hyper-reactivity was observed compared to WT mice (87); an observation that correlated with a reduction of T_REG_ cells in the lungs of ST2^−/−^ mice. Thus, although IL-33 enhances T_H_2 responses, it is not essential for the development of airway hyper-reactivity but seems to play a prominent role in T_REG_ cell homeostasis. Overall, these data are in contrast to the known *in vitro* effects of IL-33 and illustrates the multifaceted roles of IL-33 in both enhancing or dampening T_H_2 cell responses in a context-dependent manner.

### T_H_1 and T_H_17 Cell Differentiation and Function

Recent experimental evidence revealed that IL-33 plays a role in the development and maintenance of type 1 immune responses. When studying the T cell response to a systemic LCMV infection, Baumann et al. identified a prominent subset of T-bet^+^ ST2^+^ T cells within the antigen-specific memory T cell pool (21). In contrast to ST2^+^ T_H_2 cells, T_H_1 cells expressed ST2 transiently. Interestingly, after injecting LCMV-TCR specific T cells in infected mice, WT, but not Tbet- or STAT4- deficient T cells were able to express ST2, demonstrating that during strong type 1 immunity, T_H_1 cells can upregulate ST2. Furthermore, ST2^−/−^ T cells failed to expand and produce high levels of IFNγ, TNFα or IL-2 after transfer in LCMV-infected mice (21), suggesting that T_H_1 cells require ST2 in order to optimally expand and function during the course of LCMV. Interestingly, a similar observation was revealed upon influenza infection, where the rapid release of IL-33 correlated with enhanced IFNγ and TNFα production (89). In fact, IL-33 was shown to potentiate *in vitro* the action of IL-12, a STAT4 inducer, in T_H_1 cells, resulting in increased production of IFNγ (72). Similarly, CD8^+^ T cells were also shown to transiently express the ST2 receptor. IL-33 enhanced the clonal expansion of activated CD8^+^ T cells and was necessary for the effective control of LCMV infection (90). These observations demonstrate a role of IL-33 to enhance IFNγ through the action of IL-12 without affecting T_H_1 polarization (Figure 2).

Finally, a recent account suggests a possible role of IL-33 in T_H_17 cell differentiation (22). These cells express the transcription factor RORγT and release IL-17A and IL-17F. Upon anti-CD3 treatment *in vivo*, ST2 surface-expression was observed by IL-17-producing T cells in the gut (22). However, IL-33 inhibited the proliferation and pro-inflammatory cytokine production of T_H_17 cells both *in vivo* and *in vitro*. Here, contrarily to T_H_2 and T_H_1 cells, IL-33 signaling controlled the exacerbated inflammatory response by T_H_17 cells, although further work is required to understand the full extent of the role of IL-33 on these cells. In summary, many T cell subsets can respond to IL-33, making the modulation of T cells responses by IL-33 complex and context-dependent.

### IL-33-Mediated Regulation of T Cells in Infection

A way to dissect the distinct roles of IL-33 on T cells is to study its effect in distinct infectious diseases. Little is known about the role of IL-33 in human diseases, as there is currently a lack of tools to identify and follow human ST2^+^ T cells, yet important progress has been made in the field through rodent models of infectious disease. IL-33 most likely plays a key role in human disease, as evidenced by increased levels of the cytokine or its decoy receptor sST2 during both viral (91–93) and bacterial (94) infections. In rodent models, IL-33 was shown to play both protective and deleterious roles during the course of infection(95).

This is seen in models of viral infections, where IL-33 plays ambiguous roles on the T cell response. In certain cases, viral virulence is linked to enhanced IL-33 release, as observed upon infection with respiratory syncytial virus (RSV) in both human and mice (96). When mice are infected with RSV, IL-33 is rapidly released in the early phases of viral infection in the lung (97). Antibody-mediated blockade of ST2 leads to a decrease in IL-13 production and eosinophil recruitment but does not affect viral growth or clearance of RSV by type 1 immune responses (97). Concomitantly, anti-IL-33 therapy was shown to mitigate the establishment of the deleterious type 2 memory response during a Rhinovirus infection that promotes airway hyper-reactivity (98). On the other hand, IL-33 was also shown to contribute to the clearance of LCMV and Coxsackievirus-B5 systemic viral infections through enhanced T_H_1 and CD8^+^ T cell responses (90, 99).

IL-33 can also play an important role in the control and clearance of parasites. In a model of intestinal infection with *Nippostrongylus brasiliensis*, a mouse-pathogenic hookworm, clearance of the parasite and the establishment of a T cell memory response required IL-33 (100). Interestingly, IL-4^+^ T_H_2 cells–as well as high levels of IgE, basophils and mast cells responses—were readily detected in infected mice lacking ST2 (ST2^−/−^) yet insufficient IL-13^+^ T_H_2 cells and ILC2s lead to a failure to clear the parasite (100). Similarly, mice infected with *Trichuris muris or Strongyloides venezuelensis* require IL-33 signaling for the effective control of the parasite (101, 102). On the other hand, in a model of visceral *Leishmania donovani* infection, IL-33 was shown to be deleterious to the host, as it inhibited the T_H_1 response necessary for the clearance of this parasite (103). This was attributed to a skewed ST2^+^ T_H_2 immune response, as these cells accumulated in the chronic lesion of *Leishmania* (104). Similarly, lack of ST2 in mice infected with the protozoa *Toxoplasma gondii*, lead to a more severe form of encephalitis, characterized by increased levels of TNFα and IFNγ (105). Finally, lack of ST2 signaling leads to a better control of the fungus *Cryptococcus neoformans*, characterized by a significant reduction in IL-5 and IL-13 production by T_H_2 cells, but no difference in the level of expression of IFNγ and IL-17A (106). Importantly, the effect of IL-33 on the skewing of T cell responses may play a major role in predisposing to virus-induced asthma through the differentiation of pathogenic T_H_2 cells over anti-viral T cells (98). These experiments provide further evidence that IL-33 influences the function of T cells in disease and this effect is highly dependent on the target tissue of infection and type of pathogen. Furthermore, IL-33 modulates important functions in other compartments of the immune system, notably the innate immune response, which was not addressed here but contributes to the overall response against pathogens (107).

## Regulatory T Cells

T_REG_ cells are an important immunosuppressive subset of CD4^+^ T cells characterized by the expression of the transcription factor Foxp3, the key master regulator that enforces the transcriptional program global phenotype and function of T_REG_ cells (108). However, T_REG_ cells can also undergo distinct epigenetic modifications and co-express transcription factors in order to acquire effector functions enabling them to migrate, survive and suppress in inflammatory sites, particularly at mucosal surfaces (109, 110). This particular ability enables them to adapt to specific environmental conditions. IL-33 was recently identified as one of the signals involved in the maintenance of Foxp3^+^ T_REG_ cell homeostasis at mucosal sites. At the steady-state, ST2^+^ T_REG_ cells represent the majority of ST2-expressing CD4^+^ T cells and are notably found in the gut (23) and lungs (111).

### Phenotypic Characteristics of ST2^+^ T_REG_

CD4^+^ T_REG_ cells, including those found at mucosal surfaces, originate from the thymus (thymic-derived tT_REG_) or develop *de novo* from polarizing signals in the periphery (peripherally-induced pT_REG_). Both tT_REG_ and pT_REG_ cells effectively suppress innate and adaptive responses including a variety of effector T cell functions (112). Interestingly, tT_REG_ and pT_REG_ were shown to play non-redundant functions in the suppression of the adaptive immune response, as both of these subsets are required to maintain immune homeostasis in the mucosa. Although surface markers capable of distinguishing them remain poorly defined, tT_REG_ generally have a fully demethylated T_REG_-specific demethylated region (TSDR), located in the *foxp3* locus, compared to pT_REG_ cells (113, 114). Helios, a transcription factor that is prominently expressed in tTreg cells, is frequently regarded as a marker of T_REG_ cells of thymic origin (115) but is also contested (116). Both Helios^+^ and Helios^−^ T_REG_ cells isolated from the lamina propria of the gut express ST2 (23), while the vast majority of Helios^+^ T_REG_ cells express ST2 in secondary lymphoid organs and in the lungs (17), all-the-while expressing high levels of other proposed markers of tT_REG_, such as Neuropilin 1 and TIGIT (*unpublished observations*). Interestingly, the expression of Helios was recently associated with distinct T_REG_ cell functions in the periphery (117) as well as the stability of *foxp3* expression on T_REG_ cells (118). Similarly, IL-33 signaling on T_REG_ cells was shown to play an important role in enhancing the stability of Foxp3 in T_REG_ cells and is notably necessary for these cells to prevent T cell-mediated colitis (23). However, the molecular relationship between IL-33 signaling and Helios expression in T_REG_ cells remains to be understood.

On the other hand, ST2^+^ T_REG_ cells also express the transcription factor GATA3. Upon IL-33 stimulation, GATA3 is rapidly phosphorylated in T_REG_ cells (23), in turn enhancing the expression of its own receptor. Expression of GATA3, like ST2, was identified in T_REG_ cells in the gut (119) where it plays a central role in (1) the maintenance of immune homeostasis (120), (2) in the stability of *foxp3* and (3) is critical for T_REG_ cells to prevent T cell mediated colitis (119). Thus, ST2 and GATA3 follow a similar pattern of expression and play similar functional roles in T_REG_ cells, indicating a strong inter-relationship between the two in orchestrating T_REG_ adaptation in the mucosa.

Finally, the STAT5 signaling pathway can be triggered by IL-2, IL-7, IL-15, or TSLP. T_REG_ cells constitutively express high levels of the IL-2 receptor α chain (CD25), as they are highly dependent on exogeneous IL-2 for survival, function and proliferation (121, 122). In contrast, T_REG_ cells express little IL-7R outside of the thymus in human and mice (123), yet IL-7 could play a role on the expansion of T_REG_ cells at mucosal sites (124). Although there is little information on the role of IL-15 on T_REG_ cells, a recent account reveals that gut-resident T cells depend on IL-15 to enhance Foxp3 over RORγT expression and block a Th17-driven inflammatory bowel disease (125). Finally, T_REG_ cells in the lungs were recently shown to express the TSLP receptor (126). So far, however, only IL-2, in the presence of IL-33, was shown to facilitate the expression of the ST2 receptor on T_REG_ cells (23). Thus, further investigation into the role of the cytokines involved in STAT5 signaling is required.

### Role of IL-33 on T_REG_ Function

IL-33 can support many aspects of T_REG_ cell functions. IL-33 facilitates the selective expansion of T_REG_ cells *in vitro* in a MyD88-dependent manner (127, 128). Moreover, ST2^+^ T_REG_ cells show increased suppressive capacity *in vitro* and *in vivo* in the presence of IL-33 (127, 129, 130), although this was recently contested (131). However, the techniques used by these groups differed and this might provide insight into the modulation of the suppressive ability of ST2^+^ T_REG_ cells.

Moreover, *in vivo*, the increased fitness and suppressive function of ST2^+^ T_REG_ cells is also highlighted by the effect of IL-33 on the maintenance of *foxp3* expression in the gut and their ability to suppress T-cell mediated colitis (23). Concomitantly, ST2^+^ T_REG_ cells readily expand in the mucosa during the course of distinct infectious diseases (111, 129), where they resist the expression of pro-inflammatory cytokines like IFNγ, even strong polarizing conditions (129). IL-33-responsive T_REG_ cells are also endowed with unique cytokine production potential. For example, ST2^+^ T_REG_ cells were found to produce high levels of IL-10, TGFβ and amphiregulin, which favor a tolerogenic environment and the establishment of tissue repair mechanisms (111, 129) (Figure 2). On the other hand, ST2^+^ T_REG_ cells can also express type 2 cytokines, like IL-5 and IL-13, when stimulated *in vitro* in the presence of IL-33 (129). Similarly, in mice exposed to airway allergens in combination with IL-33, WT, but not ST2^−/−^, T_REG_ cells express high levels of IL-5 and IL-13 (131). Thus, there are reports of both highly suppressive and pro-inflammatory ST2^+^ T_REG_ cells. To answer this disparity, it was proposed that IL-33 could facilitate the transition from suppressive to dysregulated T_REG_ cells in a dose-dependent manner, although more investigations are required (56). On the other hand, we know little about the potential effect of secondary signals on ST2^+^ T_REG_ cells, as these cells could have acquired the ability to respond to other environmental cues.

### ST2^+^ T_REG_ in Disease

We do not know the full extent of the role of ST2^+^ T_REG_ cells in infectious diseases. Nonetheless, ST2^+^ T_REG_ cells were shown to (1) promote the establishment of memory T cells, (2) control the expansion of inflammatory T_H_1 and T_H_17 cells, and (3) promote epithelial cell repair (23, 111, 129). The role of IL-33 on T_REG_ cells has been studied in several infectious and non-infectious inflammatory models. In models that elicit prominent T_H_1 or T_H_17 responses, the role of IL-33 on T_REG_ cells was shown to be protective. For example, during Influenza infection, ST2^+^ T_REG_ cells accumulate in the lung where they produce amphiregulin, a cytokine involved in tissue repair (111). Throughout infection, ST2^+^ T_REG_ cells are refractory to inflammatory signals and resist the production of inflammatory cytokines. Moreover, in a mouse model of T-cell induced colitis, ST2 expression by T_REG_ cells was shown to be critical to prevent the onset of disease in the gut (23). Moreover, ST2^+^ T_REG_ cells are induced upon cytomegalovirus (CMV) infection in mice where they play a critical role in dampening liver damage (132). Finally, we recently observed that in chronic infection with *Cryptococcus neoformans*, which leads to a prominent T_H_17 response, ST2^+^ T_REG_ cells resist the up-regulation of RORγT and the production of IL-17 (133). However, this suppressive function of T_REG_ cells could have a negative impact, as it was shown that in helminth infections ST2^+^ T_REG_ cells, but not ST2^−^, suppress T_H_2 cells and facilitate helminth fecundity (134). Similarly, a recent account revealed that the tumor-specific release of IL-33 can promote the accumulation of T_REG_ cells at the site where they contribute to tumor growth and immune evasion (135). Thus, the effect of IL-33 was suggested to be generally protective and promote immune regulation, notably through an enhanced suppressive ability of T_REG_ cells. However, this effect seems to be context-dependent, as recent evidence reveals that IL-33 can also fuel inflammatory responses (131, 136).

### Role of IL-33 in Autoimmune Diseases

IL-33 was shown to play important roles in either driving or dampening dysregulated T cells responses in autoimmune diseases. Polymorphisms in the *Il33* gene are detected in patients with Alzheimer's disease (137) and Inflammatory Bowel disease (IBD) (138) suggesting that a complete or partial loss of function leads to exacerbated disease (139). In addition, increased levels of IL-33 are detected in patients with multiple sclerosis (MS) (140), systemic lupus erythematous (SLE) (141), type 1 diabetes (T1D) (142) and rheumatoid arthritis (RA) (143). At the steady-state, high levels of IL-33 are produced in the central nervous system (CNS), where it favors the release of IL-1β and IL-10 (144). Expectedly, IL-33 is a major component of the global inflammatory process within the CNS. In experimental autoimmune encephalitis (EAE), a mouse model for multiple sclerosis (MS), IL-33 plays a protective role by dampening the generation of inflammatory astrocytes and the expansion of effector T cells, while enhancing T_REG_ and T_H_2 responses (145). IL-33 directly attenuates the production of IL-17 and IFNγ by pathogenic T_H_17 or T_H_1 cells (146). Moreover, adoptive transfer of MOG-specific T cells from ST2^−/−^ but not ST2^+/+^ mice fail to prevent EAE onset in BALB/c mice, a strain that is naturally resistant to the disease (147). On the other hand, administration of recombinant IL-33 (rIL-33) is shown to exacerbate EAE in C57BL/6 mice while anti-IL-33 therapy attenuates IL-17 and IFNγ production *in situ* (148). This strain-specific difference may to be due to a time or context-dependent effect of IL-33, as signaling during the onset of disease is most likely protective, while IL-33 activity in the later stages likely exacerbates T_H_1 and T_H_17 responses (149).

A similarly complex role of IL-33 is found in rheumatoid arthritis (RA). While IL-33 is produced at high levels in joints during both RA in human and in experimental arthritis in mice, anti-ST2 therapy significantly attenuates the progression of disease (150). However, while ST2^−/−^ mice show reduced disease severity, IL-33^−/−^ mice do not (151), although the reasons for this difference remain unknown. Similarly, the attenuating effect of IL-33 in the onset of disease was also shown in mouse models of uveitis (152) and T1D (153), although this observation is yet to be described in human disease.

Finally, IL-33 is closely associated to asthma, since it is increased in asthmatic patients (154) and was shown to potentiate airway hyper-reactivity (136). Notably, IL-33 was shown to directly impair T_REG_ cell function during antigen-driven type 2 airway hyper-reactivity (131) and enhance T_H_2 differentiation through enhanced OX40 ligand interaction (155). Interestingly, this unexpected effect of IL-33 on T_REG_ cells differed from prior reports showing that IL-33 facilitated the suppressive function of T_REG_ cells. Future experiments will have to address these controversial observations.

### Clinical Implications of IL-33 and Related Therapeutics

The immunomodulatory functions of IL-33 are being exploited to develop novel therapeutic avenues. The IL-33/ST2 axis is currently being targeted in pre-clinical studies [reviewed by Chen et colleagues (156)]. Among the latest strategies developed to inhibit IL-33 signaling in exacerbated type 2 immune responses are monoclonal antibodies against IL-33 that mimic the capturing effect of the sST2, as they bind the biologically active IL-33 and prevent its association with the membrane receptor (157). Similarly, the use of IL-33 traps, using the extracellular domains of ST2 and IL1RAcP, and blocking the membrane-bound ST2 are strategies currently being investigated with drugs in Phase I or II clinical trials (156).

Although the rationale for the use of inhibitory drugs is mostly based on the effects of IL-33 on innate immune responses, the use of drugs or biologics that enhance the IL-33 signaling pathway generally aims to target the adaptive immune response. One notable exception is the use of IL-33 blockade in tumor microenvironments. For example, it was recently shown that monoclonal antibody blockade of IL-33 in mice xenografted with human non-small-cell lung carcinoma (NSCLC) decreased the accumulation of T_REG_ cells and reduced macrophage M2 polarization, leading to the efficient inhibition of tumor growth (158). Similarly, neutralization of IL-33 inhibits the development of colorectal cancer in mice (135), as IL-33 promotes T_REG_ cell accumulation. On the other hand, an engineered IL-2-IL-33 fusion protein was developed to reduce renal injury in mice by targeting and enhancing T_REG_ cells homeostasis and proliferation *in situ* (159). Moreover, administration of IL-33 during the recovery phase of DSS-induced colitis in mice was shown to enhance recovery, by skewing the accumulation of T_H_2 and T_REG_ cells over T_H_1/T_H_17 responses in the gut (160). Finally, it was recently suggested to use IL-33 to potentiate highly suppressive T_REG_ cells *ex vivo*, as adoptive transfer of these cells attenuates disease progression in a model of type 1 diabetes (130). However, care must be taken when considering the use of drugs that influence the IL-33 axis. For example, local IL-33 production in a mouse model of hepatocellular carcinoma was shown to enhance CD4^+^ and CD8^+^ anti-tumor activity (135), warranting a re-evaluation of the use of IL-33-neutralizing drugs in tumor models.

## Conclusion

T cell function at mucosal sites is intimately linked to the processes of antigen presentation, polarizing cytokine signaling, migration to inflamed sites and the subsequent adaptation to local conditions. The role of “alarmins” in the modulation of mucosal T cell function is yet to be fully understood. Nonetheless, IL-33 was shown to play a major role in this process, illustrating the potential for other, less studied, alarmins to play similar roles.

We focused this review on the recent advances in IL-33 and T cells, but the complexity of the relationship between the adaptive and the innate immune response dictate further investigation into the effect of IL-33 on APC-T cell activation. Notably, little is known about the effect of IL-33 on the modulation of Notch signaling, a key component of T cell differentiation.

In T cells, IL-33 plays a major role in cytokine production, cell proliferation and immune regulation. However, many aspects of T cell responses to IL-33 remain to be elucidated. Notably, many reports have shown that GATA3 and STAT5 play clear roles in promoting the transcription of *il1rl1*, yet the role of T-bet, STAT4, and STAT3 remain obscure. Thus, we need more insights into the factors that influence IL-33 signaling, from the transcription of the receptor to its effect on the function of T cells. The discovery that IL-33 could directly impact distinct T cell subset differentiation and effector functions is of particular interest, as favoring a given type of response might alter the proper course of immune control and cause irreparable damage to the host. Further investigation into co-stimulatory factors might reveal how distinct alarmins influence each other. Multiple factors may compete, synergize or otherwise influence each other in the inflammatory “soup” to which T cells are exposed to. Finally, a thorough understanding of the kinetics of each alarmin might reveal the intrinsic mechanism by which competing alarmins orchestrate the balance between inflammation and tolerance.

In summary, IL-33 can play both inflammatory and regulatory roles during the evolution of an immune response. A deeper understanding of the effects of IL-33 will undoubtedly open the door toward the generation of unique therapeutic approaches. In fact, the use of a chimeric IL2/IL33 protein was shown to be protective in renal injury (159) and monoclonal anti-IL-33 antibodies where shown to excert promising effects in the control of atopic dermatitis. (161). However, when considering a therapeutic modulation of IL-33 signaling, care must be observed in light of the multifaceted roles of IL-33.

## Data Availability

No datasets were generated in this study.

## Author Contributions

All authors listed have made a substantial, direct and intellectual contribution to the work, and approved it for publication.

### Conflict of Interest Statement

The authors declare that the research was conducted in the absence of any commercial or financial relationships that could be construed as a potential conflict of interest.
